# Food Polyphenols and Type II Diabetes Mellitus: Pharmacology and Mechanisms

**DOI:** 10.3390/molecules28103996

**Published:** 2023-05-10

**Authors:** Rabia Naz, Fatima Saqib, Samir Awadallah, Muqeet Wahid, Muhammad Farhaj Latif, Iram Iqbal, Mohammad S. Mubarak

**Affiliations:** 1 Department of Pharmacology, Faculty of Pharmacy, Bahauddin Zakariya University, Multan 60000, Pakistan; rabianazbhutta@gmail.com (R.N.); muqeetsoomro@msn.com (M.W.); farhaj@irasp.edu.pk (M.F.L.);; 2 Department of Medical Lab Sciences, Faculty of Allied Medical Sciences, Zarqa University, Zarqa 13110, Jordan; sawadallah@zu.edu.jo; 3 Department of Chemistry, The University of Jordan, Amman 11942, Jordan

**Keywords:** type II diabetes mellitus, polyphenols, resveratrol, curcumin, quercetin, catechins, hydroxycinnamic acids, anthocyanins, kaempferol

## Abstract

Type II diabetes mellitus and its related complications are growing public health problems. Many natural products present in our diet, including polyphenols, can be used in treating and managing type II diabetes mellitus and different diseases, owing to their numerous biological properties. Anthocyanins, flavonols, stilbenes, curcuminoids, hesperidin, hesperetin, naringenin, and phenolic acids are common polyphenols found in blueberries, chokeberries, sea-buckthorn, mulberries, turmeric, citrus fruits, and cereals. These compounds exhibit antidiabetic effects through different pathways. Accordingly, this review presents an overview of the most recent developments in using food polyphenols for managing and treating type II diabetes mellitus, along with various mechanisms. In addition, the present work summarizes the literature about the anti-diabetic effect of food polyphenols and evaluates their potential as complementary or alternative medicines to treat type II diabetes mellitus. Results obtained from this survey show that anthocyanins, flavonols, stilbenes, curcuminoids, and phenolic acids can manage diabetes mellitus by protecting pancreatic β-cells against glucose toxicity, promoting β-cell proliferation, reducing β-cell apoptosis, and inhibiting α-glucosidases or α-amylase. In addition, these phenolic compounds exhibit antioxidant anti-inflammatory activities, modulate carbohydrate and lipid metabolism, optimize oxidative stress, reduce insulin resistance, and stimulate the pancreas to secrete insulin. They also activate insulin signaling and inhibit digestive enzymes, regulate intestinal microbiota, improve adipose tissue metabolism, inhibit glucose absorption, and inhibit the formation of advanced glycation end products. However, insufficient data are available on the effective mechanisms necessary to manage diabetes.

## 1. Introduction

Phytochemicals and polyphenols in fruits and vegetables have antidiabetic effects [1]. Plant-based nutrients such as vegetables (onion, cabbage, and especially broccoli), fruits (apples, grapes, cherries, pears, and various berries), and grains contain hundreds of different polyphenols [2,3,4]. In this context, some vegetables such as beans, cabbage, onions, and cereals also contain anthocyanidins, whereas red fruits are the primary source of these polyphenols [5]. The plant kingdom contains a large number of polyphenols that fall under the categories of tannins, lignans, stilbenes, phenolic acids, and flavonoids, among others [6]. On the other hand, fruits, spices, grains, vegetables, and other phenolic-rich plant products contain phenolic acids (hydroxycinnamic acids and hydroxybenzoic acid), stilbenes, and lignans [3,4,7]. Phenolics are crucial to fruit quality because they impact the fruit’s taste, appearance, and nutritional value [8]. For example, flavonoids may lessen the risk of developing diabetes [6] by maintaining glucose uptake, blood glucose points, and insulin secretion, controlling immune function [9,10]. In this respect, dietary flavonoids demonstrated a significant anti-hyperglycemic-like effect through glucose absorption control [11], a reserve of digestive enzymes [12,13], regulation of intestinal microbiota [14], inhibition of the formation of innovative glycation end products [15], and other mechanisms. Polyphenols may also influence the signaling pathways and ensuing alterations in gene expression [16,17]. By controlling the events of glucose metabolism, hepatic enzymes, and lipid profiles, flavonoids reduce the pathogenesis of diabetes and its complications [18]. Flavone C-glycosides, which can also hinder digestive enzymes and activate insulin signaling, can lessen the production of advanced glycation end products (AGEs) [19]. Accordingly, the consumption of purple carrots, high in anthocyanins (flavonoids) and low in carotenoids, was linked to a decrease in impaired glucose tolerance [20]. Quercetin, a flavonoid, has received the most research attention for its in vivo and cellular anti-diabetic properties in animal and cell models [21], followed by kaempferol [22], luteolin [23], myricetin [24], and naringenin [25]. The most well-known sources of the stilbenes class of polyphenols, including resveratrol, are mulberries, grape skin, and peanuts [26]. The numerous and diverse phytochemicals known as polyphenols contain phenolic rings [9]. In this regard, two aromatic rings are joined by a 3-carbon chain to form an oxygenated heterocyclic ring, and this structure makes up a class of phenolic compounds known as flavonoids [27]. Anthocyanins, flavonols, flavones, isoflavonoids, and syringic acid are flavonoid subclasses connected to diabetes because the consumption of food that contains these compounds lowers the risk of type II diabetes [28].

According to estimates, there will likely be over 300 million cases of type II diabetes worldwide by 2030 [29]. Therefore, medical professionals, academics, and policymakers are taking note of the rising number of fatalities brought on by diabetes, related illnesses, and physiological disorders to promote healthy eating habits [1]. Currently, preventing and treating metabolic syndrome and type II diabetes involves increasing physical activity and decreasing calorie intake [30]. Hyperglycemia is a metabolic disease with multiple underlying origins that necessitate lifetime medication therapy and dietary adjustments. In diabetes management and prevention, herbal supplements are now supported by a growing body of scientific research. Nutritional polyphenols, the most common phytochemical in human diets, have drawn much interest due to growing evidence of their positive effects on humans. Dietary polyphenols aid in the management of type II diabetes and lessen the severity of diabetic complications in animals. The anti-diabetic effects of resveratrol [31,32], curcumin [33], and anthocyanins [34] have been demonstrated in humans. Studies validate that these polyphenols conducted in vitro and in vivo compounds have anti-inflammatory, antioxidant, chemopreventive, and neuroprotective properties. Accordingly, and because of the wide range of preventive and therapeutic and preventive options of food polyphenols and their involvement in managing and preventing type II diabetes mellitus, this review discusses the chemopreventive and therapeutic ability of these natural polyphenols in treating and managing type II diabetes mellitus. In addition, the current work discusses the numerous mechanisms of action through which these polyphenols exert their antidiabetic effects.

## 2. Results

### 2.1. Pathogenesis of Type II Diabetes Mellitus

**Figure 1:**
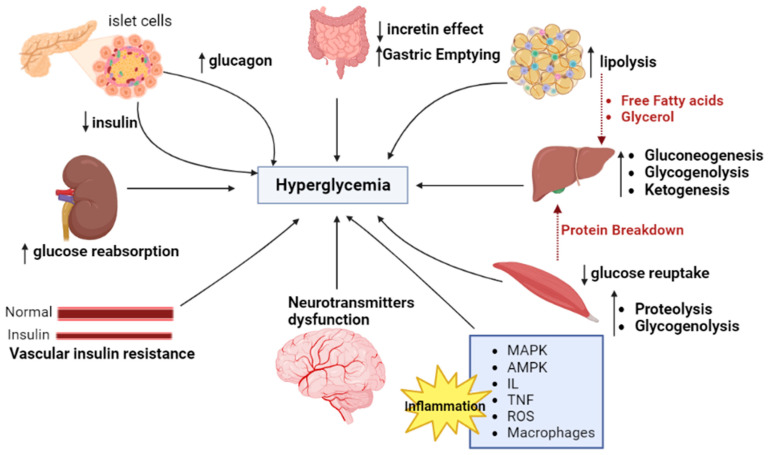
The increase or decrease in different physiological factors causes hyperglycemia.

Over 400 million people worldwide have type II diabetes (T2D), regarded as a multifactorial and complex metabolic disorder [35,36,37,38]; T2D is a chronic inflammatory disease [37]. Insulin resistance, deficiency of insulin secretion, and reduction of its anabolic activity on target tissues alter the metabolism, and its reflected chronic metabolic disorder can lead to death [39]. Through its numerous organ complications, diabetes lowers the quality of life [40] and affects whole-body physiology [41]. In this regard, hormones such as insulin and glucagon [42,43], adipokines/lipokines (adiponectin [44], leptin [45], and adipsin [46]), metabolites (amino acids [42,47], such as alanine [48,49]), lipids, free fatty acids [49,50,51], and glucagon-like peptide-1 are known metabolic regulators that disturb metabolism by signaling to various nerves and are crucial for T2D [52]. Even though T2D is most frequently passed down through families, it does so because of the interaction between risk genes primarily expressed in insulin resistance in target organs and β-cells, many other forms of hyperglycemia have nongenetic causes [53]. Depicted in Figure 1 are the essential factors attenuating type II diabetes mellitus.

#### 2.1.1. Adipokine and Pro-Inflammatory Cytokine Roles in Diabetes

An adipokine called adiponectin stimulates AMP-activated kinase (AMPK), which reduces gluconeogenesis and improves insulin sensitivity in the liver [54]. In addition to the liver, adiponectin also affects the muscles by triggering AMPK, increasing acetyl CoA carboxylase (ACC) phosphorylation, fatty acid oxidation, and glucose uptake [55,56]; adipokine aids in maintaining the homeostasis of energy [57,58]. In this context, inflammatory and metabolic diseases are complicated by the presence of molecules such as retinol-binding protein 4 (RBP4) [59], TNF-α [60,61,62], and others that interfere with homeostasis [58,59]. By producing myokines, skeletal muscles contribute significantly to the endocrine response and T2D [63]. The most well-known myokine with various functions in numerous tissues is IL-6, which is frequently linked to inflammatory processes. In a murine model, IL-6 enhanced insulin signaling via AKT while inhibiting the expression of gluconeogenic genes [64]. In addition, IL-6 increased fat oxidation and lipolysis in adipose tissue by activating AMPK [65]. IL-15 aids in enhancing insulin action and lowering visceral adipose tissue [66]. TNF-α plays a significant role in this situation because of the buildup of fat in adipose tissue due to its production and release during inflammation, which promotes insulin resistance and increases lipolysis [67,68]. To further reduce insulin sensitivity, TNF-α inhibits IRS1 and downregulates PPAR-c in adipose tissues [69,70]. The cytokines generated by NF-kB activation can stimulate JNK, which causes insulin resistance and self-activates NF-kB in a feedback loop [37]. The macrophage initiates pro-inflammatory pathways and releases TNF, IL-1b, and IL-6 [71,72,73,74,75]. The recruitment of macrophages to tissues is mediated by elevated levels of chemoattractant protein-1 (MCP1), which is part of the inflammatory response [76]. The production of monocyte chemoattractant protein-1 (MCP1) by pancreatic islets is associated with pathophysiological conditions of pancreatic dysfunction [77]. Additionally, the inflammatory response is triggered by prostaglandins and leukotrienes, which are produced from arachidonic acid. Many factors contribute to inflammation, including pro-inflammatory cytokines, ROS, and environmental factors that release eicosanoids [78,79].

#### 2.1.2. Insulin and β-Cell Involvement in Diabetes

**Figure 2:**
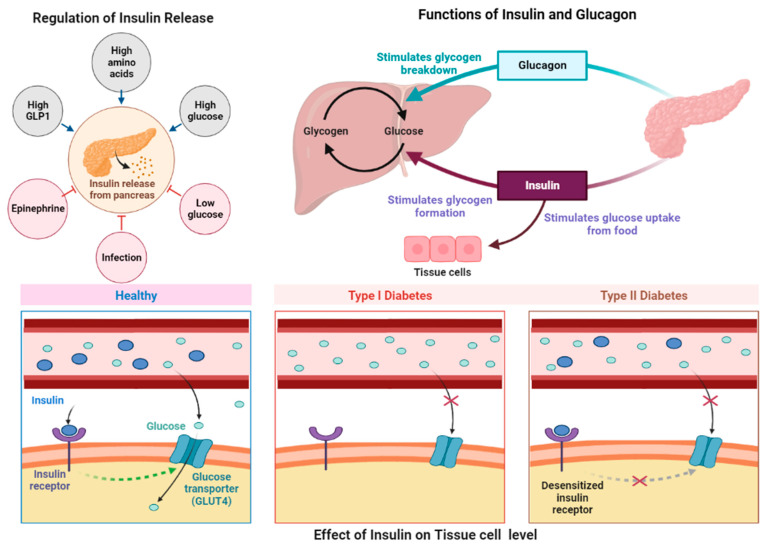
Regulation of insulin release, functions of insulin and glucagon, and effect of insulin on the healthy; regulations of type 1 and type 2 diabetes.

β-Cells are stimulated to produce and secrete insulin when the plasma glucose levels are physiological, which helps the liver, brain, muscles, and adipocyte tissue absorb glucose. Insulin prevents the breakdown of fat and promotes the synthesis of proteins, lipogenesis, and glycogen while inhibiting hepatic gluconeogenesis [80]. This proves that insulin has generalized hormonal effects in addition to its well-known ability to lower blood sugar, which explains why diabetes affects various tissues. The hormone’s binding to the insulin receptor initiates a sequence of phosphorylation events that make up the insulin signal transduction pathway. Thus, the activation of intracellular protein substrates starts signaling cascades. Afterward, phosphatidylinositol 3-kinase (PI 3-kinase) activates protein kinase B (PKB), also known as AKT. GLUT4 is then translocated to the plasma membrane, except hepatocytes, which primarily express the non-insulin-regulated glucose transporter 2 (GLUT2), where it is activated by insulin in target cells along with several other enzymes, including glycogen synthase. The mitogen-activated protein kinase pathway is also responsive to insulin signaling, which controls gene expression, protein translocation, and cell growth [81]. Because insulin is a central regulator of lipid, protein, and carbohydrate metabolism regulator, an imbalance in metabolic paths directly affects how insulin behaves. The liver’s abilities to induce glucose uptake and glycolysis, which produce the building blocks for fatty acid synthesis, are just two of the numerous mechanisms contributing to lipogenesis [82]. Production of the pancreatic enzyme is dysregulated in T2D because of the close functional connections between the endocrine and exocrine pancreas [83]. Insulin resistance develops before insulin hypersecretion, which is viewed as a step to meet high insulin requirements [84]. In this respect, insulin resistance would result in hyperinsulinism. Whatever the underlying cause of hyperinsulinemia, the result is a reduction in glucose uptake by the muscles and an increase in the production of liver glycogen, which aids in the progress of T2D [36], [85], [86,87,88,89]. Furthermore, high glucose levels can cause β-cells to express the proapoptotic receptor FAS, which can produce IL-1b [90]. Insulin and glucagon functions associated with diabetes are shown in Figure 2.

By phosphorylating FOXO1 and SREBP1, AKT2 mediates the transcriptional activation of lipogenic genes induced by insulin [91]. Nucleotides are cofactors in crucial metabolic processes in addition to carbohydrates [92], and they may be connected to metabolic diseases [93]. For example, glyoxylic acid, trimethylamine, and uridine are all upregulated in T2D [94,95,96]. Interestingly, IMP, GMP, AMP, GTP, inosine, guanosine, and adenosine levels were elevated in T2D [97,98].

#### 2.1.3. Free Fatty Acids and Type II Diabetes

Fatty acids have been linked to the risk of T2D [87]. In the blood, with increased insulin levels and insulin resistance in the liver and tissues, free fatty acids (FFAs) contribute to fat buildup, oxidative stress, inflammation, and hyperglycemia [85,86,99]. Furthermore, increased levels of FFAs prevent the lipolysis of adipose tissue induced by insulin [85]. Abnormal de novo lipogenesis and increased FFA levels are the root causes of several metabolic diseases [85,100,101,102,103]. As T2D progresses, one metabolic change occurs, which is an increase in FAAs. This change may open additional pathways that could help the disease progress. For instance, the lipid mediator palmitic acid has toxic effects in the islets, which activate the toll-like receptor to cause decreased insulin secretion and target organs’ insulin resistance [104,105]. In the liver and white adipose tissue (WAT), saturated fatty acids also cause the pro-inflammatory response via TLR4 [105,106,107]; NF-kB activation results in inflammation [108] and endoplasmic reticulum (ER) stress in immune cells and metabolic organs, which leads to insulin resistance [109,110]. Furthermore, there is a strong correlation between impaired insulin secretion and fatty acids. It has long been thought to be an aspect of the progress of type II diabetes, even though the molecular mechanisms relating to insulin resistance and fatty acids are still unknown [31]. FFAs modify islets in various techniques and accelerate the onset of T2D [36,111].

Phospholipids and triglycerides (TGs) are hydrolyzed to produce FFA and mono- and diacylglycerols (DAG), and TGs are inhaled as free fatty acids. Short- and medium-chain FFAs can be seen in the intestines, are carried to the bloodstream by serum albumin, and are stored in the liver and adipose tissues [112]. Moreover, lipogenesis is an additional source of FFAs [113]. FFAs’ high levels activate numerous pathways that may work together to affect the consequences of T2D and insulin resistance. Elevated palmitate levels induce a pro-inflammatory response by promoting IL-1 and IL-I8 secretion and maturation [114]. The serine phosphorylation of the insulin receptor substrate-1 (IRS1) in an NK/IKK-dependent fashion results in insulin resistance induced by pro-inflammatory cytokines [115]. Furthermore, high FAA concentration stresses cells because lipotoxicity causes apoptosis, ROS production, and ER stress [116]. However, sustained high-level exposure to FFAs causes lipotoxicity, which causes β-cell dysfunction and, ultimately, type II diabetes (T2D) [117]. Similarly, continual FFAs are due to the reserve of glucose-stimulated insulin (GSIS) release, changes in gene appearance, and promotion of apoptosis caused by stimulation of inaccessible pancreatic islets with stimulatory glucose concentrations [118]. ER stress, which can lead to β-cells apoptosis, can be brought on by saturated fatty acids. In β-cells, the ER stress and unfolded protein response are incredibly sensitive [119]. T2D is consequently developed in pancreatic islets exposed to FFAs over an extended period. Adipocytes can store adipose tissue more effectively when high FFA concentrations are present, but an increase in adipocyte fat content may cause inflammation and hypoxia in the tissue and cell [120].

Adipocytes develop insulin resistance and chronic low-grade inflammation, which help in the pathogenesis of T2D [116,121]. According to the most widely accepted theory, β-cells secrete too much hormone to counteract insulin resistance [117]. In this case, myocytes frequently take in more FFAs and store them as TGs because T2D increases the flux of TGs and FFAs. For metabolic energy, skeletal muscles primarily use glucose and FFAs [120,122]. FFA buildup in myocytes causes the synthesis of toxic ceramides and DAG, which can cause cell damage, lipotoxicity, inflammation, and insulin resistance [120].

Inflammation and metabolic disorders are frequently associated with metabolic dysregulation in the liver and muscles [123,124] because the accumulation of DAG promotes PKC activation while inhibiting insulin receptor activation, resulting in muscle and liver insulin resistance [125,126,127,128]. Insulin resistance positively correlates with irregular lipid buildup in the muscle and liver [129]. In this regard, elevated plasma FFA levels cause fat to build up in the WAT, liver, and muscle by regulating long-chain acyl-CoA, TGs, and DAG [128]. However, insulin resistance appears to lead to augmented lipid accumulation in these tissues [130]. Activating PKC isoforms, DAG, a precursor to TGs, regulates the phosphorylation of molecules in the insulin pathway [131]. In the development of T2D, DAG buildup appears to be a significant lipid mediator, inhibiting insulin sensitivity in the liver and muscle [130,132,133]. Furthermore, the activation of phosphatase 2A, which dephosphorylates AKT, reduces the translocation of the PIP3–PDK1 complex and inhibits insulin-stimulated AKT at the plasma membrane of target cells [134,135,136,137,138]. In addition to these mechanisms, ceramide buildup in membrane domains activates caspase, releasing pro-inflammatory cytokines, generating ROS, and leading to cell death [139].

There has been evidence linking higher levels of FFAs in people with high plasma-free radical levels to the production of ROS by NADPH oxidase in adipocytes, which led to the release of pro-inflammatory cytokines from WAT [140,141]. ROS are essential for inflammation and signaling [142]. Two tissues where pro-inflammatory cytokines may be produced and released are adipose tissue and the liver. These cytokines may affect other tissues due to blood circulation, resulting in tissue damage, cell death, and an intensified pro-inflammatory response [37]. IRS1 in the liver and adipose tissue is inhibited by lipid mediators, TNF-α, ROS, hypoxia-activated IKKb, and JNK [143,144,145]. IKK and JNK1 phosphorylate IRS1 and IRS2 on the serine residue, which causes activation of the gene linked to insulin resistance and inflammation [146,147]. On the other hand, pro-inflammatory cytokines such as IL1b, MCP1, TNF-a, and IL-6 can be produced and released when the NF-kB pathway is stimulated by high FFA concentrations [147]. Free fatty acids can bring on insulin resistance in several different ways; increased lipid metabolism caused by FFAs is linked to insulin resistance [148,149] because it inhibits the insulin receptor [150,151]. Additionally, high FFA levels cause ER stress in β-cells and the liver [152,153], as well as in adipocytes [154,155], which activates JNK and results in insulin resistance [155]. In T2D and obesity, FFAs are also necessary for the activation of the NLRP3 and the production of IL-1b [120]. IL-1 and IL-18 are released by the NLRP3 inflammasome, which promotes inflammation [154,155,156].

### 2.2. Polyphenols

**Figure 3:**
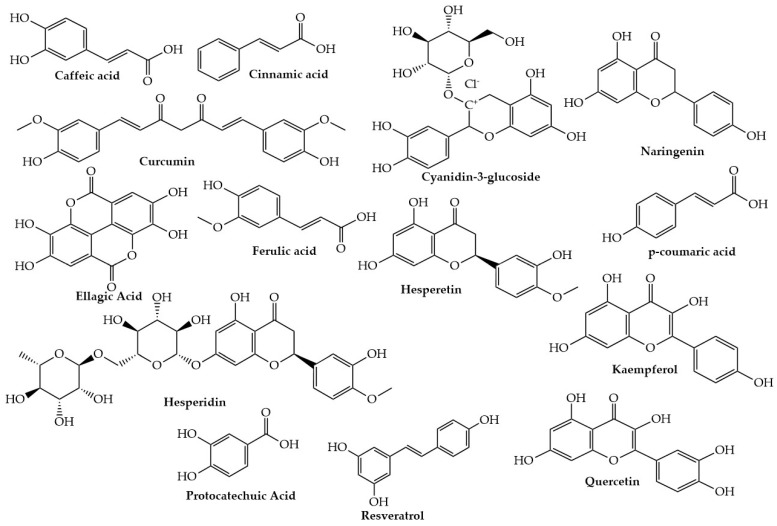
Chemical structures of polyphenols.

A growing body of evidence from in vivo and in vitro studies points to a substantial role for dietetic polyphenols in treating type II diabetes (T2D) through insulin-dependent tactics, such as protecting pancreatic islet cells, reducing cell apoptosis, promoting islet cell proliferation, attenuating oxidative stress, activating insulin signaling, and stimulating insulin secretion [33]. This can also be achieved through insulin-independent approaches including the modification of the inflammatory response, inhibition of digestive enzymes, regulation of intestinal microbiota, and prevention of advanced glycation end products from forming [120]. Plant-based foods are increasingly used in dietary guidelines for people at the hazard of T2D. These may affect glucose breakdown through several mechanisms, including carbohydrate digestion inhibition and intestinal glucose absorption, stimulation of pancreatic β-cells insulin secretion, glucose release from the liver, initiation of insulin receptors and glucose acceptance in the insulin-sensitive tissues, and modification of hepatic glucose output [2,5]. Below are details about the role of documented polyphenols in T2D. The chemical structures of some important polyphenols are shown in Figure 3.

#### 2.2.1. Resveratrol

Baur and coworkers reported that resveratrol increases the lifespan in high-caloric diet mice by reducing glucose and improving insulin levels. It increased insulin sensitivity in diabetic mice and homeostatic model assessment during glucose tolerance tests [157]. Research findings showed that resveratrol lowers blood insulin levels in animals with hyperinsulinemia and insulin resistance. Rodents with diet-induced hyperinsulinemia were used to demonstrate this effect [51,52,53,54,86]. On the other hand, resveratrol seems to raise blood insulin levels in rodent models of type II diabetes with reduced-cell mass and hypoinsulinemia, as demonstrated in db/db mice [57,61]. The improvement in insulin action lowers blood glucose levels, which prevents glucotoxicity, the harmful effects of hyperglycemia on β-cells [120]. In addition, resveratrol alleviates steatosis and lowers hepatic lipid buildup. Decreased expression of acetyl-CoA carboxylase (ACC) and fatty acid synthase (FAS) is linked to these effects [37,53,54,61,92,93,94,95]. It also reduces the expression of fatty acid synthase [156]. According to some published research, resveratrol’s effects on FAS and ACC may be mediated by the AMPK/SIRT1 axis [97,98]. It also decreases plasma amylase levels, which increases pancreatic damage. Thus, it prevents pancreatic damage.

In addition, resveratrol increases mitochondrial numbers and citrate synthase activity [158] with reduced caloric and exercise [158,159]. Furthermore, in liver tissue, resveratrol decreases the appearance of pro-inflammatory cytokines [83,94] and increases glutathione peroxidase activity, which decreases oxidative liver damage [96]. Furthermore, resveratrol decreases inflammatory markers, which protect pancreatic β-cells [103]. Findings also demonstrated that resveratrol lessens oxidative stress; reduces islet fibrosis and destruction; restores islet architecture; enhances islet structure and function; and attenuates other worsening changes in db/db mice, a type II diabetes animal model with diminished β-cell mass. Moreover, resveratrol increases the β-cell mass and partially stops β-cell failure [57,61]. Parametric analysis of gene set enrichment (PAGE) showed that resveratrol alters glycolysis, TCA cycle, classic and alternative complement pathways, butanoate, propanoate metabolism, and sterol biosynthesis [157]. In insulin-resistant rodents, resveratrol promotes intracellular glucose transport in rats fed a high-cholesterol and high-fructose diet and given resveratrol larger than those animals not given this supplement [160]. Resveratrol enhances skeletal muscle’s ability to absorb insulin-stimulated glucose [161,162].

##### Resveratrol Effect on Diabetes via GLUT4 Elevation

In insulin-resistant rodents, intracellular glucose transport increases by resveratrol. Within this context, Deng and colleagues indicated that when rats fed on a high-fructose and high-cholesterol diet are given resveratrol in the initial animal studies, they show greater soleus muscle glucose uptake than animals not given this supplement [160]. Similar results were obtained and showed that resveratrol increases skeletal muscle glucose uptake in rats nourished on a high-fat diet [161,163]. Resveratrol increases intracellular glucose transportation in insulin-resistant animals via two GLUT4-related mechanisms. It is well recognized that resveratrol expedites the translocation of GLUT4 to the muscle cells’ plasma membranes [160,161], and GLUT4 expression is also increased in animals with insulin resistance in their skeletal muscle [164] and in db/db mice [165]. Moreover, research findings showed improved insulin action by increased intracellular glucose transportation in resveratrol-consuming insulin-resistant animals. In skeletal muscle, resveratrol reduces insulin resistance through various mechanisms, including alterations in metabolism and lipid buildup. In addition, resveratrol encourages mitochondrial biogenesis in rats with diet-induced insulin resistance in their skeletal muscles [166] and improves mitochondrial β-oxidation [162]. Coen and Goodpaster reported that type II diabetes and insulin resistance are exacerbated by increased intramyocellular lipid accumulation, affecting how well insulin works [167].

##### Resveratrol Effect on Diabetes via SIRT1 Involvement

Kitada et al. [168] reported that variations in the expression and activities of two intracellular controllers are closely related to the beneficial effects of resveratrol on the muscle tissue of insulin-resistant rodents, i.e., SIRT1 and AMPK. The NAD+-dependent histone deacetylase SIRT1 (silent information regulator 1) involves several processes, including inflammation, mitochondrial biogenesis, stress resistance, intracellular metabolism, glucose homeostasis, apoptosis, and others. Since type II diabetic patients have decreased SIRT1 activity and expression, SIRT1 is considered a target for anti-diabetic medications [168,169]. In addition, scientists showed that resveratrol triggers SIRT1 in mammalian tissues [170] and triggers muscle SIRT1 in animals with diet-induced insulin resistance [162]. An increase in the NAD+/NADH ratio is related to this enzyme’s activation [166]. Findings also revealed that resveratrol raises the SIRT1 level in the muscle in rodents with genetically stimulated insulin resistance [56]. Deacetylation and activation of PGC-1α are linked to resveratrol-induced upregulation of AMPK in skeletal muscle, possibly via SIRT1-dependent mechanisms [164,168].

##### Resveratrol Effect on Diabetes via AMPK Activation

Another enzyme involved in the action of resveratrol, besides SIRT1, is AMP-activated protein kinase (AMPK). AMPK controls various physiological functions, such as mitochondrial function, energy metabolism, insulin secretion, and biogenesis [171]. In this regard, McCart reported that AMPK promotes insulin sensitivity and fatty acid oxidation [172]. Furthermore, resveratrol activates AMPK by phosphorylation and acetyl-coA carboxylase [158]. Insulin resistance induced by the diet in animal models is preceded by decreased AMPK activity [82], and insulin resistance is genetically determined [54]. The insulin-sensitizing medicines thiazolidinediones and metformin usually stimulate AMPK in various tissues, even though a direct connection between AMPK initiation and the reduction of insulin resistance in humans has not been established [171]. Resveratrol activates AMPK to these drugs in insulin-resistant animals. Resveratrol also reverses diet-induced insulin resistance in rodents by restoring AMPK phosphorylation [51] and makes AMPK active in skeletal muscle [165].

##### Resveratrol Effect on Diabetes Involving Mitochondria

Resveratrol reduced the acetylation status of PGC-1α [157], a transcriptional co-activator that regulates the mitochondrial biogenesis mediated by SIRT1 deacetylation [173,174]. In addition, it is believed that in humans, mitochondrial muscle dysfunction speeds up intramuscular lipid deposition and reduces insulin action [64]. Therefore, resveratrol action in muscle tissues appears to depend on the rise in mitochondrial biogenesis caused by a concurrent reduction in intramuscular lipid level [168,169].

##### Resveratrol Effect on Diabetes via FFA Reduction

Increased release of free fatty acids is identified as a significant factor in the emergence of insulin resistance [100,101] in rodents [50,54,83] with diet-induced insulin resistance. In this respect, resveratrol has been shown to lower pancreatic triglyceride levels in animals fed with high-fat diets [52]. The anti-obesity properties of resveratrol may be connected to its anti-diabetic properties [13,14], with decreased action of lipogenic enzymes (acetyl-CoA carboxylase, glucose-6-P-dehydrogenase, and lipoprotein liPase) [92]. It is well known that having more body fat reduces the effectiveness of insulin and increases the risk of developing type II diabetes in humans [2,44]. Without causing appreciable changes in adiposity, resveratrol may enhance insulin action [55] or decrease body weight [56,83]. By increasing insulin receptor phosphorylation, resveratrol may also enhance insulin signaling in animals with insulin resistance in their skeletal muscles [39] and increased protein levels of IRS-1 [56]. Table 1 shows the antidiabetic activity of resveratrol from molecular mechanisms to in vivo studies.

#### 2.2.2. Curcumin

Curcumin (Figure 3) exhibits anti-inflammatory properties that may aid in controlling diabetes. Curcumin analogs have been identified and are currently the subject of extensive research for their potential roles in diabetes. In this regard, numerous studies on the effectiveness of curcumin in regulating blood glucose in various rodent models have been published. According to Arun and Nalini, curcumin lowers blood sugar, hemoglobin (Hb), and glycosylated hemoglobin levels (HbA1C) [188] and recovers insulin sensitivity [189]. Similarly, Abu-Taweel and coworkers reported that curcumin improves diabetes pathology through various mechanisms, including the control of lipid metabolism; antioxidant activity; and other activities such as antiapoptotic, anti-inflammatory, and antihyperglycemic activities [190]. Research findings indicated that curcumin extract reduces insulin resistance, prevents cell death, delays the onset of diabetes, and enhances cell functions in animal models [191]. Similar results were obtained when 250 mg curcuminoids were used for nine months in pre-diabetic patients not diagnosed with diabetes. Furthermore, Chuengsamarn et al. [33] reported that curcumin improves the overall performance of β-cells with higher homeostasis model assessment (HOMA-β) and lower C reactive protein (CRP). Those who received curcumin experienced higher levels of adiponectin and lower levels of insulin resistance. In the meantime, Wickenberg reported that postprandial serum insulin concentrations increased by 6 g turmeric ingestion without having an appreciable impact on plasma glucose levels [192]. A paper by Gutierres and colleagues showed that giving curcumin for 31 days to STZ-induced diabetic rats reduced the hyperlipidemic and hyperglycemic effects [193]. On the other hand, a different study found curcumin (90 mg/kg BW) with insulin (1 U/day vs. 4 U/day) in STZ-induced rats decreased hyperglycemia, hypercholesterolemia, and biochemical markers of kidney and liver damage while increasing the activity of glutathione peroxidase and superoxide dismutase (hepatic antioxidants) [194].

In addition, curcumin has excellent wound-healing qualities due to its capacity to reduce oxidative stress by removing free radicals [195]; many people with diabetes experience difficulties with wound healing [196]. In this context, Yang and coworkers showed that curcumin can prevent retinal attenuation by enhancing the retina’s ultrastructure [197]. By promoting the superoxide dismutase enzyme’s expression, curcumin can reduce oxidative stress [198] and the reduction of ROS production, both of which are crucial for treating diseases such as diabetes caused by oxidative stress and inflammation [199]. Oxidative stress is thought to make diabetes worse, whereas ROS have been proposed to be crucial in diabetes pathogenesis. Curcumin’s chemical makeup and anti-oxidative strength allow it to function naturally as a free radical scavenger. Fasting blood glucose (FBG), hemoglobin A1c (HbA1C), estimated average glucose (EAG), and body mass index (BMI) levels were all improved by curcumin in diabetic patients [200]. In this respect, Panahi et al. reported that curcuminoid supplementation has an antioxidant effect in T2DM patients because it reduced malondialdehyde (MDA) and raised serum SOD activity and total antioxidant capacity [201]. Similarly, Jain reported that curcumin diet supplements (50 or 100 mg/kg BW) decrease hyperglycemia and inflammatory processes in STZ-induced diabetic rats by preventing McP-1, HbA1c, TNf-α, IL-6, and lipid peroxidation and suppressing the NF-kB signaling pathway; protecting against inflammation [202]; and restoring normal antioxidant enzymes levels, including catalase, glutathione peroxidase, and SOD [203].

He et al. [204] also reported that curcumin prevents the NF-kB signaling cascade and inflammation. Reduced levels of IL-6 and TNF-a were assessed in STZ-induced diabetic rats with heart damage in a study by Abo-Salem et al. [205]. On the other hand, Arafa showed that curcumin could increase insulin sensitivity by decreasing cholesterol and blood glucose levels [206]. A high curcumin supplement (100 mg/kg) improved insulin intolerance and glucose in gestational diabetes mice by triggering the AMPK pathway [207]. Findings also showed that curcumin treatment significantly decreased superoxide production and NADPH oxidase subunit expression (p67phox, p22phox, and gp91phox) in diabetic rats. This effect may have been caused by curcumin inhibiting the protein kinase C (PKC)-MAPK signaling pathway [208]. Oxidative stress and endoplasmic reticulum (ER) were protected from diabetes by the novel curcumin analog C66, which inhibited JNK activation in diabetes [209]. Additionally, results showed that curcumin significantly increased mitochondrial permeability and decreased palmitate-induced oxidative stress. It did this by causing pancreatic β-cells to secrete more insulin when glucose was present [210]. Pathological complications of diabetes include diabetic nephropathy, diabetic neuropathy, vessel damage, and cardiovascular diseases [211]. In contrast, Panahi et al. [212] reported that taking curcumin (1 g daily) for three months reduces leptin levels and the leptin/adiponectin ratio (an indicator of atherosclerosis) in patients with atherosclerosis; it also increased adiponectin. Table 2 shows data related to the antidiabetic activity of curcumin.

#### 2.2.3. Quercetin

Quercetin (Figure 3) has been proven useful in treating T2D [230]. Research by Pereira and coworkers showed that quercetin interacts with molecular marks in the adipose tissue, liver, skeletal muscle, pancreas, and small intestine to maintain glucose homeostasis [231]. Other studies reported that quercetin treats T2D by reducing hyperglycemia, enzyme levels, liver glucose content, high blood pressure, serum cholesterol levels, and hyperlipidemia, as well as by encouraging weight loss [230,232], lowering blood sugar levels [233,234,235], improving glucose tolerance [233,236] and hepatic glucokinase activity [236], and enhancing the subsequent release of insulin and pancreatic cell regeneration [237,238]. In this respect, research findings revealed that quercetin activates AMPK, which inhibits glycogenic isoenzymes such as phosphoenolpyruvate carboxylase (PEPCK) and glucose-6-phosphatase (G6Pase) to reduce glucose synthesis [235,239] and stimulate protein kinase B (Akt) and skeletal muscle GLUT4 receptors, which in turn activates AMPK in the cell membrane [240]. Pereira confirmed that the GLUT4 transporter controls blood sugar levels by controlling glucose entrance into the cells [231]. In another study, Borghi indicated that by encouraging the GLUT4 translocation to the cell membrane, quercetin administration, GLUT2 expression, and intestinal-sodium-dependent glucose uptake are reduced, thus lowering gastrointestinal absorption of glucose and controlling blood sugar levels [241].

Similarly, Spínola et al. showed that the inhibition of pancreatic-amylase and intestinal-glucosidase decreases starch hydrolysis, slows postprandial hyperglycemia progression, and diminishes the rate of glucose absorption by quercetin usage [242,243]. Another study reported that quercetin improves dyslipidemia caused by a high-fat diet (HFD) in Swiss albino mice [244]. By controlling the levels of c-peptide and HbA1c, quercetin reduced the harm to pancreatic β-cells [245] and decreased lipid levels and insulin resistance [246], thus increasing pancreatic β-cell functions and exerting anti-hyperglycemic activity in diabetic rats [247]. In this respect, 20 µM of quercetin induced a significant increase in insulin secretion by increasing intracellular calcium ions through interaction with L-type Ca^2+^ ion channels in INS-1 β-cells [248], as well as simultaneous transient inhibition of KATP channels [249]. According to these results, quercetin controls glucose metabolism by enhancing glycolysis and reducing gluconeogenesis [250]. Moreover, published research showed that fat accumulation, reduced body weight, dyslipidemia, hyperglycemia, and hyperinsulinemia were significantly improved by quercetin treatment due to improved gene-associated glucose or lipid metabolism in high-fat-fed obese mice [246,251]. In addition to lowering blood sugar and HbA1c levels, Wang et al. found that oral administration of quercetin in multiple doses improved glycogen synthesis, decreased insulin resistance, and lowered glucosidase activity. Furthermore, it decreased oxidative stress, which enhanced pancreatic insulin secretion and helped diabetic patients control their blood glucose levels [209]. In addition, quercetin helps in alleviating diabetic complications by blocking AR [252].

The protein expression of insulin-signaling molecules such as phosphatidylinositol 3-kinases (PI3K) and insulin receptor substrate-1 (IRS-1) can be increased by quercetin, according to studies on STZ-induced diabetic rats; this results in an increase in insulin-mediated glucose uptake [231]. A survey by Ashraf and colleagues showed that quercetin lowers oxidative stress by scavenging ROS and improving the AMP/ATP ratio in clonal pancreatic cells [253]. On the other hand, obesity-related T2DM is associated with fat buildup in the muscles and liver, which triggers the nuclear transcription factor NF-B (NF-B) and Jun N-terminal kinase (JNK) inflammatory pathways [254]; both of these pathways are suppressed by quercetin [255]. In addition, brown adipose tissue releases pro-inflammatory mediators such as IL-8, IL-4, IL-1, IL-6, TNF-α, and histamine in response to high blood glucose levels and improved insulin resistance [256]. These mediators are inhibited by quercetin, which also reduces oxidative stress [257]. Blocking the enzymes lipoxygenase and cyclooxygenase prevents the release of pro-inflammatory mediators such as prostaglandins and leukotrienes [258]. Yao et al. reported in a clinical survey conducted among the Chinese population an inverse relationship between quercetin consumption and the prevalence of T2D [259]. Table 3 lists the antidiabetic activity of quercetin and its mechanisms of action.

#### 2.2.4. Catechins

Kim and colleagues reported that catechins stimulate either GLUT4 transcription or translocation to the plasma membrane in muscle cells and glucose uptake in peripheral tissues. Furthermore, catechins inhibit lipogenesis, glycogen synthesis, and glucose oxidation in liver cells [290]. Similar results were reported by several studies [291,292,293,294,295]. Catechins can also impair glucose transporters on the plasma membrane of intestinal cells, Similarly, epicatechin gallate inhibits the Na^+^-dependent glucose transporter in rabbit intestinal brush-border membrane vesicles (SGLT1), demonstrating that epicatechin gallate inhibits SGLT1 [296,297]. Moreover, researchers showed that catechins prevent weight gain and the start of chronic illnesses such as T2D or metabolic syndrome when consumed regularly [298,299]. Similarly, other researchers indicated that epigallocatechin gallate inhibits pancreatic glucosidase in a noncompetitive manner that is reversible [300,301,302]. Moreover, galloylated catechins are more potent than nongalloylated catechins at inhibiting glucosidase and amylase. Depending on their chemical composition, catechins have varying levels of inhibitory power [303].

#### 2.2.5. Isoflavones

Findings showed that the consumption of isoflavone decreased the risk of diabetes [304] via glucose uptake inhibition and negligible intestinal carbohydrate absorption [305]. In addition, isoflavones enhance insulin sensitivity and resistance, safeguarding pancreatic β-cells, acting as an anti-inflammatory agent, reducing oxidative stress, and preventing the formation of the Maillard reaction and advanced glycation end products [306]. In this context, Rockwood et al. reported that genistein significantly lowers hyperglycemia in T2D [307,308], increases cell proliferation while decreasing apoptosis [309], and reduces oxidative stress and cardiac inflammation [310]. In contrast, daidzein’s preventive effect on reducing hyperglycemia, dyslipidemia, obesity, insulin resistance, inflammation, and other T2D complications has been thoroughly studied. It causes an immunomodulatory effect in mice with diabetes [311,312]. To incorporate several methods to increase flavonoids’ antidiabetic activity, numerous strategies have been developed in recent years to use flavonoids in vitro and in vivo models.

#### 2.2.6. Hydroxycinnamic Acids

##### Ferulic Acid

Published research revealed that ferulic acid (FA) lowers hyperglycemia, the lipid profile, creatinine, urea, serum glutamic oxaloacetate transaminases, and serum glutamic pyruvic transaminases while maintaining islet mass in STZ-induced diabetic rats over the course of three weeks [313]. At doses of 0.01 and 0.1% of the standard diet, FA lowered blood glucose levels in STZ-induced diabetic mice. In KK-Ay mice, 0.05% FA significantly lowered blood glucose levels [314]. Similarly, oral administration of FA (10 and 50 mg/kg BW) into STZ-induced diabetic rats demonstrated antioxidant activity; it decreased the levels of lipid peroxidation indicators in the serum, liver, pancreas, and kidney [315]. In this respect, several food items such as tomatoes, berries (such as strawberries), rice husks, and other fruits and vegetables commonly contain FA [316,317]. By increasing plasma insulin levels, glucokinase activity, and liver glycogen synthesis in diabetic rats, FA and sinapic acid effectively decreased blood glucose levels [318,319].

##### Gallic Acid

Gandhi et al. reported that gallic acid (GA) exhibits antidiabetic properties in animal models lacking insulin or are resistant to insulin [320] by significantly reducing blood sugar, triglyceride, total cholesterol, urea, uric acid, low-density lipoprotein cholesterol, and creatinine while simultaneously raising plasma levels of insulin (16.3 U/mL), C-peptide, and glucose tolerance [321]. Other researchers showed that GA reduces gluconeogenesis and increases glycolysis, ultimately decreasing hyperglycemia in STZ-induced diabetic rats [322]. Fruits such as grapes and berries contain GA [323,324]; in this regard, researchers found that apple juice and berries might help improve short-term glycemic control [9].

##### Protocatechuic Acid

Protocatechuic acid (PCA) showed reduced levels of hepatic gluconeogenic enzymes such as fructose-1,6-bisphosphatase, glucose 6-phosphatase (G6Pase), and sorbitol dehydrogenase, as well as increased levels of glucose-6-phosphate dehydrogenase and hexokinase in STZ-induced diabetic rats [325]. These results show that PCA can enhance GLUT4 translocation, adiponectin secretion, and glucose uptake [326]; prodigious amounts of PCA are found in gooseberry, raspberry, blueberry, mulberry, honey, soybeans, and loquat fruit [325].

##### Ellagic Acid

Ellagic acid (EA) might be a useful dietary supplement to lessen the metabolic changes associated with HFD feeding animals in combination with STZ injection [327]. EA reduces glycation stress, hyperglycemia, inflammation, and hyperinsulinemia and aggravates renal function dose-dependently. In this respect, research findings showed that EA (3.12–50 M) increases the expression of PPAR in L6 myotubes and GLUT4 [328].

##### Salicylic Acid

Blackberries, cantaloupes, blueberries, dates, grapes, apricots, kiwis, olives, green peppers, radishes, tomatoes, and mushrooms are among the foods that contain salicylic acid in high concentrations. This acid lowers blood concentrations in diabetic Goto-Kakizaki rats [329].

##### Caffeic Acid

Numerous fruits and vegetables, including blueberries, kiwis, cherries, plums, apples, pears, potatoes, artichokes, cider, and coffee, contain caffeic acid (CA), a phenolic acid [7]. Researchers reported that dietary supplements with CA (0.02% in the diet for five weeks) decrease blood glucose, G6Pase, and phosphoenolpyruvate carboxy kinase activities, accompanied by a decrease in the liver GLUT2 expression and enhanced insulin levels, glucokinase, catalase, glutathione peroxidase, and SOD activities in db/db mice [330]. Additionally, CA significantly lowered the levels of plasma HbA1c [331]. In insulin-resistant rats undergoing a glucose test, administration of CA reduced the elevation of plasma glucose levels. CA also increases the isolated adipocytes’ ability to absorb glucose. Moreover, the reduction in plasma glucose appears to be caused by CA’s increased glucose utilization [332].

##### *p*-Coumaric Acid

Another phytochemical, *p*-coumaric acid, is prevalent in fruits and vegetables, including apples, pears, beans, potatoes, tomatoes, tea, and pineapple [333,334,335]. By changing glucose and lipids’ metabolism, *p*-coumaric acid can potentially prevent or treat insulin resistance and T2D [336].

##### Chlorogenic Acid

Chlorogenic acid (CGA) increases GLUT in skeletal muscle by phosphorylating AKP-activated protein kinase, which enhances the metabolism of lipids and glucose, thus reducing the hazard of diabetes [337]. Evidence suggests that CGA reduces intestinal-sodium-gradient-driven glucose transport and inhibits G6Pase. It increased AMPK phosphorylation and favorable metabolic changes linked to AMPK activation while improving skeletal muscle glucose uptake and lipid profiles [338]. In addition, Bassoli and coworkers reported that inhibiting G6Pase activity prevents the production of hepatic gluconeogenesis [339]. Moreover, it reduced hepatic steatosis and inhibited the expression and activity of G6Pase in the liver [340]. Cherries, apples, kiwis, artichokes, eggplants, plums, and coffee are just a few of the foods that contain CGA, one of the most prevalent phenolic compounds [7]. CGA reduces the effects of retinopathy and other diabetic complications in animals by preventing retinal neo-angiogenesis [341]. Furthermore, enzymes that break down carbohydrates are weakly inhibited by chlorogenic acid [342]. Research findings indicated that CGA inhibits glucosidase activity [343].

##### *trans*-Cinnamic Acid

*trans*-Cinnamic acid (t-CA) is found in numerous food-related plants, fruits, and herbs [344]. Through the involvement of GLUT4, t-CA (1 ng/mL) isolated from Cinnamomum cassia activates insulin-mediated glucose transport [345]. In isolated islets, it significantly increased glucose-enhanced insulin secretion [346]. Daily oral administration of t-CA (80 mg/kg BW) for four weeks decreased hyperglycemia in male albino rats with diabetes induced by alloxan [347]. These results demonstrate that treatment with t-CA (80 M) increases AMPK activation and adiponectin secretion. Additionally, the inhibitory effect of paclitaxel suggests that t-CA-stimulated signaling in 3T3-L1 adipocytes involves a G-protein-coupled receptor and enhances insulin sensitivity [348].

#### 2.2.7. Anthocyanins/Anthocyanidins

Zhou and coworkers reported that anthocyanidins (ACNs) promote health through their antioxidant, anti-inflammatory, and blood-sugar-regulating properties [235]. In this regard, AMPK/ACC/mTOR pathway helps anthocyanin-rich mulberry extract prevent hyperglycemia [349]. Other researchers showed that by managing blood lipid and triglyceride levels, lowering cholesterol, and having low-density cholesterol while raising high-density cholesterol and apolipoprotein, ACNs might reduce insulin resistance [350]. Moreover, anthocyanins stimulated the release of insulin by increasing the appearance of the intracellular Ca^2+^ signaling pathway and the glucose-transport-related gene (Glut2) in mouse islet β-cells. Along this line, purple potato extract with added cyanidin increased insulin secretion [351]. Delphinidin 3-arabinoside anthocyanidins, found in fermented berry beverages, controlled DPPIV and its substrate GLP-1, boosted insulin secretion, and increased the mRNA expression of genes related to insulin receptors [352]. Published work by Graf et al. showed that ACN-rich grape-bilberry juice (AGBJ) supplementation improved several risk factors for diseases linked to obesity in male Fischer rats for ten weeks. Results revealed that AGBJ intervention successfully reduced serum levels of triglycerides and leptin while having no impact on the release of adipokines, adiponectin, glucose, insulin, or non-esterified fatty acids. In addition, AGBJ increased plasma levels of polyunsaturated fatty acids while lowering levels of saturated fatty acids. Overall, the findings suggested that AGBJ might effectively combat metabolic diseases linked to obesity [353]. In STZ-induced T2DM rats, ACNs from purple root vegetables reduced liver damage and oxidative stress and enhanced lipid and blood glucose levels [354].

ACNs act as anti-inflammatory agents by suppressing the expressions of a few inflammatory cytokines crucial to the inflammatory response, including TNF-, IL-6, and IL-1 [355,356,357,358]. Monocyte chemoattractant protein 1 (MCP-1), a chemokine, plays a role in developing diabetes mellitus by controlling leukocyte migration and infiltration [359]. Numerous studies demonstrated that ACNs can lower MCP-1 expression [358,360]. In addition, research findings showed that ACNs could be a potent therapeutic agent to prevent obesity and diabetes because of the changes in AMP-activated protein kinase activation. ACNs decreased the AMP/ATP ratio, which strongly correlated with ACN supplementation. [361]. AMP-activated protein kinase (AMPK) is a critical molecule in the control of glucose metabolism in the liver, white adipose tissue, and skeletal muscle, which is activated by ACNs [354,362,363,364,365]. Activation of AMPK induces GLUT4, thus improving glucose utilization and uptake [365,366]. Moreover, the production of the liver’s glucose is decreased when AMPK is activated [367]. Findings confirmed that ACNs could help with obesity, as well as impaired glucose tolerance, insulin resistance, and DM prevention. Cyanidin-3-glucoside (C3G) improved glucose tolerance (GT) and reduced body weight gain in mice fed with a high-fat diet [368]. In this regard, numerous studies demonstrated that ACN-rich blueberries can decrease body weight, enhance lipid profiles, suppress the countenance of inflammatory factors, and increase insulin sensitivity in animal models fed with a high-fat diet [356,369,370,371]. Black elderberry [360], raspberry [372], Aronia melano-carpa [373,374], and black rice [375] are rich in ACN and could improve insulin resistance and lipid metabolism in the liver or serum in obese mice.

Takikawa et al. [362] reported that bilberry extract containing an increased ACN level significantly decreases blood glucose levels in T2DM mice and improves insulin sensitivity. Feeding T2DM mice a diet containing 0%, 5%, or 10% buckwheat sprouts revealed that as the number of buckwheat sprouts in the diet increases, lipids levels and blood glucose improve more noticeably [376]. Similarly, ACNs from the black soybean seed coat could also lessen the harm done to the liver, kidney, and pancreas in STZ-induced T2DM mice [377]. In a different experiment involving animals, giving blueberry ACN extract to T2DM mice improved glucose tolerance and blood glucose levels; reduced polydipsia and polyuria symptoms; and reduced TC, TG, and insulin levels [378]. Ye and colleagues reported that C3G intervention reduces blood sugar and insulin resistance and improves blood sugar and lipid parameters in db/db mice [379]. Furthermore, diabetic db/db mice supplemented with dietary C3G for 5 weeks showed reduced hepatic triglyceride content and steatosis and decreased inflammatory cytokine concentration in the serum [380].

On the other hand, malvidin and ACNs were used in combination with metformin in the treatment of STZ-induced diabetic rats, and the outcomes demonstrated that the combination therapy has more significant relief from insulin resistance, decreased fasting blood glucose, and improved lipid metabolism and serum insulin compared to single therapy [381]. After receiving combined treatment with fenofibrate and ACNs in T2DM patients with postprandial hyperlipidemia, the serum postprandial triglyceride level and LDL cholesterol concentration were pointedly reduced (from black soybeans) [382]. Several studies showed that ACNs can decrease the initiation of pro-inflammatory factors and improve insulin resistance [367,383]. ACNs prevent the stimulation of JNK and NF-B, which lowers the phosphorylation of IRS-1 serine residues and improves insulin resistance [367,371]. Additionally, it has been demonstrated that ACN can trigger the production of adiponectin, which can potentially reduce insulin resistance [358,384,385]. ACNs increase the efficiency of two enzymatic antioxidants called SOD and catalase (CAT), which shield cells from oxidative damage by catalyzing the conversion of free radicals into hydrogen peroxide [358,386]. Furthermore, the inflammatory response may accelerate the development of DM complications and contribute to insulin resistance, eventually resulting in T2D complications [387]. Cranberries, blackberries, chokeberries, black grapes, gooseberries, bilberries, red raspberries, blueberries, blackcurrants, and strawberries are rich sources of ACNs. Other sources include a variety of other fruits such as peaches, grapes, nectarines, pomegranates, plums, cherries, seeds, and vegetables, i.e., red onions and red lettuce [388]. Table 4 lists the anthocyanins’ role as potential antidiabetic agents along with their molecular mechanisms.

#### 2.2.8. Kaempferol

Kaempferol exhibits anti-oxidative stress anti-hyperglycemic [394], anti-inflammatory [395], and hypolipidemic [396] effects. Inflammatory cytokines, including TNF-α and IL-6, stimulate the c-Jun amino-terminal kinase (JNK) and I-kB kinase-b/nuclear factor-kB (NF-kB) paths in insulin-sensitive organs and inhibit insulin signaling [397]. Similar to an insulin secretagogue, kaempferol enhances insulin secretion. Kaempferol increased plasma insulin levels while lowering the blood glucose level in STZ-induced diabetic rats [398]. Kaempferol directly activates mitochondrial calcium uptake (MCU) in a concentration-dependent manner. An amount of 1 µM can trigger the pancreatic β-cell secretion/metabolism/coupling and closely dual the uptake of mitochondrial Ca^2+^ [399,400]. With an increase in cAMP, Ca^2+^, and glutathione (GSH) levels, kaempferol raises glucagon-like peptide 1 (GLP-1) and insulin levels [401]. In this respect, Fang et al. showed that in 3T3-L1 adipocytes, kaempferol enhances insulin-dependent glucose uptake [402]. Kaempferol also lowers blood glucose levels by boosting GCK levels and enhancing glycogen synthesis [22].

An imbalance in the making and utilization of glucose leads to disorders of glucose metabolism. Hepatic IR plays a significant role in fasting hyperglycemia. In this regard, abnormal glucose-metabolism-regulating enzyme levels, such as phosphoenolpyruvate carboxykinase, PC, glucokinase (GCK), and glucose-6-phosphatase, are a hallmark of hepatic IR (PEPCK). Blood sugar levels directly affect how GCK is activated and inactivated. Activation of GCK is thus a probable target for diabetes treatment [403]. Kaempferol (50 mg/kg/day), administered orally to mice, significantly reduces hyperglycemia by reactivating hexokinase and inhibiting PC and gluconeogenesis [394]. A direct rise in the activity of Akt and inhibition of PC are additional components of the mechanism by which kaempferol inhibits hepatic gluconeogenesis [22], as Akt phosphorylates and suppresses FOXO1 transcription when insulin signaling is activated, ultimately suppressing PEPCK and G6P expression [404,405]. As part of its anti-inflammatory effects, kaempferol prevents the hepatic inhibitor IkB kinase/NF-kB pathway and restores Akt activity [406]. To create phosphatidylinositol (3,4,5)-triphosphate, insulin first binds to the insulin receptor on the cell’s outer surface, causing tyrosine phosphorylation of the insulin receptor substrate (PIP3). Protein kinase C (PKC) and P70 ribosomal S6 kinase (S6K) are both activated by PIP3 after Akt, a 3-phospholipid-dependent protein kinase I, is activated [407].

The physiological effects of insulin are significantly influenced by Akt-dependent phosphorylation. GSK3a/b is first inactivated by Akt-induced phosphorylation, which then causes dephosphorylation and activation of glycogen synthase [408]. To control the intracellular GLUT4 vesicle movement to the cell membrane and boost glucose uptake, Akt phosphorylates the 160 kDa TBC1D4/AS160 substrate [409,410]. To have an anti-inflammatory effect, kaempferol constrains the hepatic Ik-B kinase/NF-kB pathway and increases Akt activity [406]. Adipose tissues, the liver, and the muscles exhibit increased AMPK and ACC phosphorylation in response to kaempferol [411,412]. For the treatment of diabetes, AMPK activation is an important pharmacological target. In this context, thiazolidinediones (TZDs) and metformin have been recognized as AMPK activators [413]. Foods high in kaempferol can lower postprandial glucose levels and decrease carbohydrate absorption. Changes in the intestinal microbiota play a significant role in metabolic syndrome, type II diabetes, and obesity [414]. Additionally, kaempferol decreases the relative richness of thick-walled flora, boosts bacteroides, lowers blood lipid and glucose levels, and enhances IR in C57BL/6 obese mice [415]. The excellent autophagy enhancer kaempferol reduces ER stress, promotes intracellular lipid degradation, and guards against lipotoxic damage to β-cells [416]. To maintain intracellular balance, autophagy is well-defined as an intracellular lysosomal degradation process of defective proteins, macromolecules, damaged organelles, and toxic aggregates [417]; disorders of autophagy are linked to IR, obesity, and T2DM [418]. In another study, Varshney and coworkers reported that through AMPK mTOR signaling, treatment with 10 µM kaempferol increased lipid droplet co-localization with lysosomes and autophagosomes in cells and decreased ectopic lipid buildup and ER stress [419]. Chronic hyperglycemia in diabetes eventually destroys the mitochondrial function, activates nicotinamide adenine dinucleotide phosphate oxidase, and increases the production of ROS [420]. The excellent antioxidant effect of kaempferol can prevent excessive ROS from damaging β-cells. Kaempferol protects pancreatic β-cells from oxidative damage in diabetes [421]. In the kidney, liver, heart tissues, and erythrocytes of diabetic rats, kaempferol significantly increases membrane-bound ATPase activity [422]. This is yet another way that kaempferol protects β-cells. Natural plants such as ginkgo biloba, galangal, and pueraria have been used for a long time, especially in Asia, and are good sources of kaempferol. In addition, it can be found in foods such as tomatoes, beans, gooseberries, grapes, cabbage, cauliflower, and strawberries [423]. Listed in Table 5 are data pertaining to the role of kaempferol as a potential antidiabetic agent from molecular mechanisms to in vivo studies.

#### 2.2.9. Hesperetin

Hesperidin effectively reduces pancreatic β-cell dysfunction and programmed cell death in diabetic rat models, as well as the expression of the 78-kDa glucose-regulated protein (GRP78) [429]. Additionally, by upregulating the anti-apoptotic cell lymphoma extra-large (Bcl-xL) and downregulating the BCL2-linked X-protein, hesperidin as an apoptosis regulator successfully modulated the expressions of apoptosis regulatory proteins (Bax) [429]. Additionally, by controlling AMPK-mediated p300 inactivation, hesperetin and naringenin protected pancreatic β-cells in both in vitro and in vivo models [430]. The apoptosis of pancreatic β-cells is influenced by the initiation of the MAPK and FoxO1/PPAR signaling pathways [431] and may accelerate the development of type II diabetes and insulin resistance [432]. Furthermore, phosphorylation of the MAPK activates NF-kB, causing the release of pro-inflammatory cytokines [433]. Research findings indicated that hesperetin metabolites reduce inflammation by preventing the phosphorylation of NF-B and MAPK. Finally, it is worth mentioning that hesperidin is most prevalent in citrus fruit [434].

## 3. Discussion

A diet high in vegetables and fruits offers several nutritional advantages. Vegetables and fruits contain polyphenols in addition to minerals, vitamins, and fiber [435,436]. Flavonoids are polyphenols, which include flavonols, flavanols, flavones, flavanones, anthocyanidins, and isoflavones. They are found in the human diet, such as in citrus fruits, which have the highest concentration of flavanones [437,438]. Increasing the intake of foods high in flavonoids has been linked to positive health effects and a decline in the incidence of chronic ailments such as type II diabetes (T2D), cardiovascular illnesses, and dyslipidemias [437,439]. By lowering oxidative stress, increasing insulin secretion, and enhancing insulin sensitivity, flavonoids protect against high glucose levels [440]. Previous research claimed that flavonoids prevent pancreatic β-cells from undergoing apoptosis [441] and that they engaged in anti-inflammatory, anti-apoptotic, and antioxidant-like activities. Flavonoids regulate these effects by modulating the activity of signaling cascades such as nuclear factor kappa-B (NF-kB) and protein mitogen-activated kinases (MAPKs) [442]. Flavonoids in these functional foods and phytomedicine have beneficial effects on immune function, blood sugar levels, glucose metabolism, and insulin secretion [9]. Numerous controlled studies showed that dietary phenolic consumption reduces diabetes risk factors by regulating the major pathways for carbohydrate metabolism and hepatic glucose homeostasis. Consuming many polyphenols is linked with a decreased risk of developing diabetes mellitus [6]. One of the phenolic acid’s best-known effects on the metabolism of carbohydrates is its ability to inhibit the key enzymes, glucosidase, and amylase, which convert dietetic carbohydrates to glucose [9,443]. Despite a genetic predisposition, dietary changes and augmented physical activity may delay the onset of type II diabetes [444,445].

Diets high in polyphenols can help in managing type II diabetes. The prevention of diabetes in various models of insulin resistance is recognized from changes in the liver, adipose tissue, and skeletal muscle, and animal studies consistently show that resveratrol improves insulin action. Resveratrol alters established pathways for aging, transforms obese mice’s physiology into that of mice on a standard diet, and enhances health, as demonstrated by various indicators such as survival, motor function, organ pathology, insulin sensitivity, PGC-1 activity, and mitochondrial number. Notably, none of these changes arose in tandem with a significant loss in body weight [157]. This is significant and indicates the potential of resveratrol to treat various diseases such as type II diabetes linked to impaired insulin action. However, human studies are needed to assess resveratrol’s therapeutic value given that type II diabetic patients may use it. It is important to note that resveratrol’s positive effects on β-cells were also observed in type II diabetic patients, significantly lowering blood insulin levels in those with hyperinsulinemia. A concurrent decline in the homeostasis model of assessment for β-cell function (HOMA-) was observed in conjunction with this effect [108]. Although resveratrol had some positive effects on type II diabetic patients, other studies showed that it did not affect blood insulin levels or HOMA-B [106]. Reduced demand for insulin is a benefit of resveratrol-induced reduction in insulin resistance. Consequently, β-cell failure is also decreased because they secrete less insulin.

Curcumin is potentially used for the treatment of diabetes and associated complications. It is an inexpensive drug and relatively safe, and it reduced hyperlipidemia and glycemia in rodent models of diabetes. Due to deficiencies in insulin secretion and its action, diabetics cannot effectively metabolize glucose, and curcumin can have a therapeutic effect by playing a crucial role in β-cell functions. A rise in blood glucose levels is a hallmark of T2DM, a heterogeneous and chronic metabolic sickness caused by insulin resistance in target tissues and pancreatic β-cell dysfunction. Preclinical research using animal models and clinical trials found that curcumin pointedly lowers fasting plasma glucose and glycated hemoglobin (HbA1c) levels, according to T2DM results. In the treatment of metabolic syndrome, curcumin successfully lowers triglycerides and LDL-C (low-density lipoprotein cholesterol); enhances fasting blood sugar levels and insulin resistance (HOMA-IR); and reduces AST levels, body weight, and aminotransferase levels [223]. Curcumin has been shown in preclinical studies to lessen inflammation by preventing and regulating the tissue release of pro-inflammatory cytokines, such as IL-4, IL-8, IL-6, and TNF-α [446].

Oral glucose tolerance and insulin secretion by pancreatic β-cells are both enhanced by quercetin. Due to its inhibition of glucosidase and DPP-IV enzymes, glucagon-like peptide-1 (GLP-1) and glucose-dependent insulinotropic polypeptides have a longer half-life (GIP). Additionally, quercetin inhibits the production of pro-inflammatory molecules such as IL-4, IL-6, IL-1, and TNF-α. Through the hangup of glucosidase and interference with glucose transport across intestinal cells’ plasma membrane, catechins regulate glucose absorption through two distinct mechanisms. In particular, catechins help to recover insulin sensitivity, lower blood lipid levels, decrease white fat depots, and reduce blood sugar and lipid levels. In vivo tests using substances such as streptozotocin and alloxan or diets (high fructose and fat diets) inducing T2D in animal models have demonstrated vital anti-hyperglycemic activity for several significant hydroxycinnamic acids, including *p*-coumaric acid, cinnamic acid, ferulic acid, caffeic acid, chlorogenic acid, and rosmarinic acid [447].

Anthocyanidins are of great nutritional interest because they have demonstrated antidiabetic activity primarily through inhibition of oxidative stress, insulin secretion promotion, insulin resistance improvement, lipid and glucose metabolism, and antioxidant and anti-inflammatory functions. One of the reasons anthocyanins have an anti-T2D outcome is because of their antioxidant properties. This is because oxidative-stress-related cell damage is a significant factor in the development of T2D. To decrease lipo-toxicity, kaempferol regulates lipid metabolism, enhances IR, and improves insulin signaling. It also restores the equilibrium between glucose production and consumption, reducing glucose toxicity. To protect β-cells, kaempferol corrects the imbalance in autophagy and apoptosis. Flavanones can improve health by changing the expression of genes and proteins in pancreatic cells. However, little is known about how flavanones work in pancreatic β-cells underneath high glycemic stress in physiologically relevant concentrations or how they affect the expression of all proteins. Citrus flavonoid hesperetin (Hst), which is effective in preventing diabetes and its complications, has recently attracted the attention of researchers. Novel methods with few side effects are urgently needed to treat diabetes and its complications. New monomeric molecules derived from herbal medicine, a type of complementary medicine, are being sought after for the cure of diabetes as well as its complications.

## 4. Materials and Methods

### 4.1. Literature Search and Methodology

In the current review on food polyphenols and type II diabetes mellitus, relevant references published between 2000 and 2022 were obtained from different bibliographical databases such as Google Scholar, PubMed, Web of Science, Science Direct, and Scopus. In our search, we used keywords related to food polyphenols (fruits and vegetables) and their pharmacologic profiling including “nutritional polyphenols”, “traditional medicinal uses”, “in vivo, in vitro anti-diabetic activities“, and “preclinical and clinical studies”. In this work, articles were chosen on the basis of the following criteria: fruits and vegetables containing polyphenols in the evaluation of in vitro/in vivo antidiabetic activity. After the selection of raw material, the pharmacology of anti-diabetic polyphenols was provided. We did not impose language restrictions in our search; however, we only included articles published in English for further consideration.

### 4.2. Illustrations and Figures

The chemical structures were drawn in ChemDraw 22.0.0 with the help of Pubchem (the mechanistic illustrated figures were drawn in Biorender (https://biorender.com/, accessed on 18 March 2023). Previously published literature data were used to draw the illustrated Figures.

## 5. Conclusions

T2D, which has a multifactorial pathology, affects millions of people around the world. Treatment of this disease includes lifestyle modifications, dietary adjustments, physical activity, and therapies involving medications for the rest of one’s life. This review article has summarized most of the in vivo and in vitro studies conducted so far to show how food polyphenols affect T2D. Recognized benefits of resveratrol in experimentally insulin-deficient diabetic animals include anti-hyperglycemic action and pancreatic β-cell protection. Curcumin is a safe and cost-effective natural anti-inflammatory and anti-diabetic property that provides a treatment option for this condition, according to several in vivo and in vitro studies, because it is pharmacologically safe, efficient, and with few side effects. In addition to their capacity to influence gene expression and glucose metabolism pathways such as AMPK, anthocyanins also have beneficial effects on insulin resistance; lipid metabolism; glucose metabolism; the immune system; and the ability to modulate hyperlipidemia, hyperglycemia, overweight, obesity, and cardiovascular diseases. Kaempferol may significantly improve how diabetes and its complications are managed. Consequently, dietary polyphenols could be used to prevent and treat diabetes. In addition, results obtained from this review show that natural ingredients are crucial for maintaining good health. Moreover, clinical studies and early research showed that polyphenols can reduce insulin resistance, blood glucose levels, and dyslipidemia in diabetic patients. More preclinical and clinical trials along with cytotoxicity tests should be conducted before these phenolic compounds hit the market as antidiabetic agents. “Over-the-counter” (OTC) polyphenol supplements for diabetics will be clinically effective because they are safe and reduce inflammation and diabetes stress.

## Figures and Tables

**Table 1:** Antidiabetic activity of resveratrol in in vivo studies with its molecular mechanisms.

Resveratrol Dose	Duration	Modal	Mechanism of Action	Ref.
5 mg	Twice a day 4 weeks	T2D patients	Decreased insulin resistance	[175]
10 mg/day	4 weeks	RCT double-blind 19 men with T2DM 55 ± 9 years	No changes in insulin levels, Tendency to decrease HOMA-IR	[175]
50 mg	Twice a day 60 days	T2D patients	No change in insulin resistance Decreased blood glucose levels Decreased diabetic ulcer size	[112,176]
75 mg/day	12 weeks	Nonobese women (with normal glucose tolerance)	Does not cause any changes in insulin sensitivity, plasma inflammation markers, and systolic blood pressure	[177]
100 mg/day	8 weeks	RCT parallel-blind 24 subjects with diabetic food Age: 56 ± 9 years old	Non-significant decrease in glucose in both study groups; no changes in HOMA-IR and insulin	[178]
150 mg	30 days	Obese men	Decreased systolic blood pressure, insulin resistance, plasma inflammation markers, and blood glucose levels	[179]
150 mg/day	30 days	Obese men	Decrease postprandial glucagon responses	[32]
150 mg/day	4 weeks	16 subjects with T2DM RCT double-blind cross-over	Non-significant changes in glucose and insulin levels, HbA1c level	[180]
200 mg/day	24 weeks	110 subjects with T2DM RCT double-blind	Significant decrease in glucose and HbA1c (*p* = 0.005), and significantly reduced insulin and HOMA-IR levels (*p* = 0.001)	[176]
250 mg/day	3 months	57 subjects with T2DM RCT open-label	Significant decrease in HbA1c (*p* < 0.05)	[181]
250 mg/day	6 months	57 subjects with T2DM RCT open-label	Nonsignificant decrease in HbA1c and glucose levels	[182]
250 mg	3 months	T2DP	Decreased blood glucose levels and systolic blood pressures	[181]
250 mg per day	8 weeks	Healthy aged men	No changes in metabolic and inflammatory status in skeletal muscle	[183]
500 mg/day	3 months	60 subjects with T2DM and albuminuria RCT double-blind	Improvement in HOMA-IR and a significant decrease in insulin, glucose, and HbA1c levels (*p* < 0.05)	[184]
500 mg	Twice a day 45 days	T2DP	Decreased insulin resistance, blood glucose levels, HOMA-β, and systolic blood pressure	[185]
500 mg 3 times a day	4 weeks	Obese men	No changes in insulin resistance, plasma inflammation markers, and systolic blood pressure	[186]
500 mg 3 times a day	90 days	Patients with metabolic syndrome	Decreased insulin resistance, but did not cause changes in systolic blood pressure	[31]
1 g/day	45 days	64 subjects with T2DM RCT double-blind	Caused a significant decrease in glucose, insulin, and HbA1c levels (*p* < 0.05), and improvement in HOMA-IR after RV administration	[185]
First week 1 g/day second week 2 g/day	2 weeks	Obese men	No change in insulin resistance and blood glucose levels Caused a decrease in the production of intestinal and hepatic lipoprotein	[111]
1, 1.5, 2 g/day	4 weeks	Older adults	Decreased insulin resistance	[110]
3 g/day	8 weeks	Overweight or obese men with nonalcoholic fatty liver disease and IR	No change in insulin resistance	[113]
3 g/day	3 months	10 subjects with TD2M RCT double-blind	Caused a decrease in HbA1c No significant changes in HOMA-IR No changes in glucose and insulin levels	[187]

**Table 2:** Antidiabetic activity of curcumin along with molecular mechanisms.

Curcumin Dose	Duration	Model	Mechanism of Action	References
0.01–1 µM	24 h	Streptozotocin-induced diabetic rats	Decreased TNF-α, IL-6, HbA1c, lipid peroxidation, and MCP-1 secretion	[202]
2.5 or 10 M	for 30 min	High-glucose-treated H9C2 cardiomyocytes	Decreased TNF-a and IL-6 (pro-inflammatory cytokines) and VCAM-1 and ICAM-1 (adhesion molecules) expressions Inhibited the HG-induced increase in fibrotic genes (collagen-IV, TGF-b, and collagen-I), and decreased AKT phosphorylation	[213]
2.5, 5, or 10 µM	once every two days for 12 weeks	Primary cultures of neonatal rat cardiomyocytes	Decreased JNK phosphorylation	[214]
0.75%	8 weeks	db/db mice	Decreased PPAR-γ via AMPK activation and decreased lipid peroxidation	[203]
10 mg/kg/day	42 days	STZ-induced diabetic C57BL/6 mice	Suppressed hyperglycemia-induced inflammation, hypertrophy, and fibrosis, and decreased TNF-α and ICAM-1	[213]
20 mg/kg	45 days	Streptozotocin-induced rats fed with a high-cholesterol diet (HCD)	Decreased glycemia and dyslipidemia	[215]
30–90 mg/kg	31 days	Streptozotocin-induced diabetic rats	Anti-hyperglycemic and anti-hyperlipidemic effect Decreased blood glucose and lipid levels, and lowered levels of hepatic antioxidants	[193,194]
0.05 g/100 g diet	10 weeks	Streptozotocin-induced rats fed with a high-cholesterol diet (HCD)	Decreased glycemia and dyslipidemia	[216]
50, 150, or 250 mg/kg	7 weeks	Streptozotocin-induced rats fed with a high-cholesterol diet (HCD)	Decreased glycemia and dyslipidemia	[217]
80 mg/kg	60–75 days	Streptozotocin-induced rats fed with a high-cholesterol diet (HCD)	Decreased glycemia and dyslipidemia	[218]
80 mg/kg	45 days	STZ-induced diabetic rats	Decreased blood glucose Decrease antioxidant defenses	[219]
100 mg/kg	28 days	Streptozotocin-induced rats fed with a high-cholesterol diet (HCD)	Decreased glycemia and dyslipidemia	[220]
100 or 200 mg/kg/day	8 weeks	STZ-induced diabetic Wistar rats	Decreased inflammatory factors (TNF-α and IL-1β) Activated AKT/GSK-3β signaling pathway	[221]
120 mg/kg	1 month	Diabetic male rats	Decreased glucose level and mitochondrial dysfunction Increased antioxidant defense	[222]
150 mg/kg,	45 days	Diabetic male rats	Decreased blood glucose and HbA1c Increased plasma insulin, AST, and ALT	[223]
0.2 g/kg	6 weeks	Diabetic db/db mice	Decreased SREBP1c, ChREBP, CPT1, and ACAT	[224]
200 mg/kg/day	6 weeks	STZ-induced diabetic Wistar rats	Inhibited IL-6 and TNF-α levels	[205]
200 mg/kg	16 weeks	Streptozotocin-induced diabetic rats	Decreased Bcl-2 Increased Bax and caspase-3	[221]
250 mg/day	9 months	240 prediabetic subjects n = 120 placebo group n = 120 curcuminoid group	0% T2DM incidence in the treated group vs. 16.4% incidence in the placebo group Increased HOMA-β and adiponectin levels Decreased HOMA-IR (insulin resistance) Decreased C-peptide level Improved β-cells function	[33,225]
300 mg	8 weeks	67 T2DM patients: n = 21 placebo group n = 22 atorvastatin group n = 23 NCB-02 group	Improved the endothelial function Decreased malondialdehyde, endothelin-1, IL-6, and NF-α	[226]
500 mg/day plus 5 mg/day for	3 months	100 T2DM patients: n = 50 in the placebo group n = 50 in the curcuminoids group	Decreased blood glucose level, C-peptide, HbA1c, alanine aminotransferase, and aspartate aminotransferase	[227]
475 mg	10 days	8 T2DM patients treated with glyburide (5 mg)	Decreased LDL, VLDL, and triglycerides Increased HDL Improved glycemic control (lower blood glucose levels after breakfast, lunch, and dinner)	[228]
1000 mg/day + 10 mg/day	12 weeks	100 T2DM patients: n = 50 placebo group n = 50 curcuminoids group	Decreased leptin and TNF-α Decrease leptin/adiponectin ratio Decreased adiponectin	[212]
300 mg/day	3 months	100 overweight/obese T2DM patients, n = 50 placebo group and n = 50 in the curcuminoid group	Decreased fasting glycemia Decreased HOMA-IR (insulin resistance) Decreased HbA1c Increased lipoprotein lipase activity Decreased FFA and triglycerides	[34,229]

**Table 3:** Antidiabetic activity of quercetin with its molecular mechanisms.

Quercetin Dose	Duration	Model	Mechanism of Action	References
10 mg/kg	4 weeks	STZ-induced diabetic rats	Decreased blood glucose and increased insulin secretion Decreased blood glucose levels Decreased creatinine and blood urea nitrogen levels	[260,261,262]
10 mg/kg	28 days	STZ-induced diabetic rats	Increased insulin secretion Decreased blood glucose levels inhibited apoptosis	[263,264]
15 mg/kg	25 days	STZ-induced diabetic rats	Decreased blood glucose levels and Improved glucose tolerance	[265,266]
20–50 mg/kg	6 weeks	STZ-induced diabetic rats	Decreased inflammation Reduced blood glucose levels Decreased fasting blood glucose Decreased hypertension Increased insulin secretion Decreased ROS production	[267,268]
25–75 mg/kg	28 days	STZ-induced diabetic rats	Increased insulin secretion and decreased blood glucose	[269]
50 mg/kg	30 days	Alloxan-induced diabetic rats	Inhibited α-glucosidase activity and reduced oxidative stress	[270]
50 mg/kg	7 days	Alloxan-induced diabetic mice	Decreased blood glucose Increased insulin secretion Decreased inflammation	[271,272]
50 mg/kg	12 weeks	HFF obese rats	Reduced oxidative stress	[270,273]
50 mg/kg	8 weeks	STZ-induced diabetic rats	Decreased blood glucose Decreased fasting blood glucose Decreased inflammation Suppressed IL-1β, TNF-α, and production of AGEs Increased insulin secretion	[274,275,276]
50 mg/kg	4 weeks	Alloxan-induced diabetic rats	Lowered blood glucose levels Decreased inflammation Decreased fasting blood glucose Increased insulin secretion Decreased creatinine, AST, ALT, and cholesterol levels	[277,278,279]
50 mg/kg	12 weeks	STZ-induced diabetic rats	Decreased the production of reactive oxygen species (ROS) and improved glucose tolerance	[280,281]
50–80 mg/kg	45 days	STZ-induced diabetic rats	Reduced blood glucose levels Improved oxidative stress Decreased LDL and VLDL cholesterol Decreased blood glucose Increased insulin secretion	[282,283]
90 mg/kg	10 weeks	STZ-induced diabetic rats	Decreased oxidative stress Decreased lipid peroxidation Reduced AGE product activity	[284,285]
100 mg/kg	14 days	STZ-induced diabetic rats	Increased insulin secretion Decreased fasting blood glucose Decreased blood glucose	[286]
100–200 mg/kg	6 weeks	STZ-induced diabetic rats	Improved glucose tolerance Decreased blood glucose Increased insulin secretion Increased HDL cholesterol Decreased triglycerides, VLDL, LDL, and total cholesterol	[287,288,289]
1 g/kg	1 month	STZ-induced diabetic Wistar rats	Improved insulin secretion insulin and increased glucose uptake Decreased fasting blood sugar	[252]

**Table 4:** Antidiabetic activity of anthocyanins and their molecular mechanisms.

Anthocyanins Dose	Duration	Model	Mechanism of Action	References
320 mg/day	4 weeks	T2D patients	Decreased FBG, LDL-cholesterol, IL-6, IL-18, and TNF-a Increased IL-10 and adiponectin (anti-inflammatory markers)	[38]
160 mg	24 weeks	T2D patients	Increased antioxidant capacity and decreased insulin resistance	[385]
1.5 mL/kg	After 12 h of fasting condition	T2D patients	Decreased FBG level, improved insulin resistance and β-cell functions	[389,390]
0.47 g	3 weeks	T2D patients	Decreased postprandial glycemia	[385]
320 mg/day	12 weeks	160 pre-diabetics, double-blind	Caused moderate reductions of LDL-c, HbA1c, apo A1, and apo B	[391]
150, 300, or 600 mg/day	4 weeks	23 healthy subjects, double-blind	Decreased glucose in the blood and hindered the secretion of insulin and incretins.	[392]
1050 mg/day whortleberry extract (9 mg anthocyanins)	2 months (every week 3 days)	37 T2D, double-blind	Decreased blood glucose levels and HbA1c	[393]

**Table 5:** Antidiabetic activities of kaempferol, along with molecular mechanisms.

Kaempferol Dose	Duration	Model	Mechanism of Action	References
0.01, 0.1, 1, and 10 µM	4 days	Human islet (CMRL-1066) cells	Decreased apoptosis and increased pancreatic β-cells	[424]
1, 10, and 25 µM	Treated on days 3, 8, and 12, and observed after 48 h of the last treatment	Human mesenchymal stem cells (hMSCs)	Decrease adipogenesis and Increased lipolysis	[425]
5, 10, and 20 µM	15 days	Zebrafish	Decreased triglyceride synthase	[426]
5 mg/kg 15 mg/kg	6 weeks	Male TSOD and TSNO mice	Decreased lipid synthesis, decreased fatty acid oxidation, and increased liver cholesterol transport	[427]
50 mg/kg	12 weeks	Male C57BL/6J mice	Decreased hepatic gluconeogenesis, increased glycogen synthesis, and decreased blood glucose	[22]
75, 150, or 300 mg/kg	8 weeks	Male Wistar rats	Increased fatty acid oxidation	[428]
100 mg/kg	45 days	Male Wistar rats	Increased membrane-bound ATPases, and increased antioxidants	[398]
200 mg/kg	8 weeks	C57BL/6 mice	Decreased blood glucose and insulin resistance Regulated intestinal flora	[415]

## Data Availability

Not applicable.

## Abbreviations

ACC	Acetyl-CoA carboxylase
ACNs	Anthocyanins
AGBJ	Anthocyanins-rich grape-bilberry juice
AGEs	Advanced glycation end products
AKT	Protein kinase B
ALT	Alanine aminotransferase
AMP	Adenosine monophosphate
AMPK	AMP-activated kinase
Apo A1	Apolipoprotein AI
Apo B	Apolipoprotein B
AST	Aspartate aminotransferase
Bcl-2	B-cell lymphoma 2
BMI	Body mass index
C3G	Cyanidin-3-glucoside
CA	Caffeic acid
CGA	Chlorogenic acid
ChREBP	Carbohydrate-responsive element-binding protein
COX-2	Cyclooxygenase-2
CPT1	Carnitine palmitoyltransferase I
DAG	Diacylglycerol
DPPIV	Dipeptidyl peptidase-4
EA	Ellagic acid
EAG	Estimated average glucose
ER	Endoplasmic reticulum
FA	Ferulic acid
FAS	Fatty acid synthase
FBG	Fasting blood glucose
FFAs	Free fatty acids
FOXO1	Forkhead transcription factor FKHR
GA	Galic acid
G6Pase	Glucose 6-phosphatase
GCK	Glucokinase
GLP-1	Glucagon-like peptide-1
GLUT2	Glucose transporter type 2
GLUT4	Glucose transporter type 4
GMP	Guanosine monophosphate
GSH	Glutathione
GSIS	Glucose-stimulated insulin
GT	Glucose tolerance
GTP	Guanosine triphosphate
HbA1c	Hemoglobin A1C
HDL	High-density lipoprotein
HOMA-IR	Homeostatic Model Assessment for Insulin Resistance
ICAM-1	Intercellular adhesion molecule 1
IKK	Inhibitor of nuclear factor-κB (IκB) kinase (IKK)
IKKb	Inhibitor of nuclear factor kappa-B kinase
IL-6	Interleukin-6
IMP	Inosine monophosphate
IR	Insulin resistance
IRS1	Insulin receptor substrate 1
IRS-1	Insulin receptor substrate 1
JNK	C-Jun N-terminal kinase
LDL	Low-density lipoprotein
MCP1	Monocyte chemoattractant protein-1
MDA	Malondialdehyde
mTOR	Mammalian target of rapamycin
NADPH	Nicotinamide adenine dinucleotide phosphate
NF-kB	Nuclear factor kappa- B
NM	Not mentioned
PC	Pyruvate carboxylase
PCA	Protocatechuic acid
PDK1	3-Phosphoinositide-dependent protein kinase-1
PEPCK	Phosphoenolpyruvate carboxykinase
PGC-1α	Peroxisome-proliferator-activated receptor-gamma coactivator (PGC)-1alpha
PI 3-kinase	Phosphatidylinositol 3-kinase
PIP3	Phosphatidylinositol (3,4,5)-trisphosphate
PKC	Protein kinase C
PPAR-c	Peroxisome proliferator-activated receptor-C
PPAR-γ	Peroxisome proliferator-activated receptor gamma
RBP4	Retinol-binding protein 4
ROS	Reactive oxygen species
S6K	S6 kinase
SGLT1	Sodium-glucose transporter 1
SIRT1	Silent information regulator 1
SOD	Superoxide dismutase
SREBP1	Sterol regulatory element-binding proteins
SREBP-1	Sterol regulatory element-binding protein 1
STZ	Streptozotocin
T2D	Type II diabetes
TAG	Triacylglycerol
*TC*	Total cholesterol
TCA	Tricarboxylic acid
TG	Triglycerides
TGF-β	Transforming growth factor-beta
TLR4	Toll-like receptor 4
TNF-α	Tumor necrosis factor α
VCAM-1	Vascular cell adhesion molecule 1
VLDL	Very low density lipoprotein
WAT	White adipose tissue
